# The physiology of singing and implications for ‘Singing for Lung Health’ as a therapy for individuals with chronic obstructive pulmonary disease

**DOI:** 10.1136/bmjresp-2021-000996

**Published:** 2021-11-11

**Authors:** Adam Lewis, Keir Elmslie James Philip, Adam Lound, Phoene Cave, Juliet Russell, Nicholas S Hopkinson

**Affiliations:** 1 Department of Health Sciences, Brunel University London, London, UK; 2 National Heart and Lung Institute, Imperial College London, London, UK; 3 Patient Experience Research Centre, Imperial College London, London, UK

**Keywords:** complementary medicine, emphysema, perception of asthma/breathlessness, respiratory muscles

## Abstract

Singing is an increasingly popular activity for people with chronic obstructive pulmonary disease (COPD). Research to date suggests that ‘Singing for Lung Health’ may improve various health measures, including health-related quality-of-life. Singing and breathing are closely linked processes affecting one another. In this narrative review, we explore the physiological rationale for ‘Singing for Lung Health’ as an intervention, focusing on the abnormalities of pulmonary mechanics seen in COPD and how these might be impacted by singing. The potential beneficial physiological mechanisms outlined here require further in-depth evaluation.

## Introduction

Singing has become an increasingly popular approach for people with chronic respiratory disease and chronic obstructive pulmonary disease (COPD) in particular.^1–3^ Various types of singing are being used within groups, including those living with respiratory disease, from community choirs with minimal disease-specific content adaptation to ‘Singing for Lung Health’ (SLH). ‘SLH’ incorporates breathing and vocal exercises comparable with those used by respiratory and speech and language therapists to support optimum breathing and vocalising.^4–7^ The British Lung Foundation has previously trained approximately 120 singing leaders to run ‘SLH’ groups, and prior to the COVID-19 pandemic, 65 groups were run by these leaders in the UK.^8^ Rather than a focus on learning repertoire for performance, a typical SLH session would integrate physical and vocal warm-ups, breathing exercises, relaxation and carefully chosen vocal repertoire all to support breath control. The performance of these components has previously been evaluated, establishing intervention fidelity.^9^ In SLH, techniques aim to have physical benefits such as improving the use of respiratory and postural muscles, flexibility, reduced hyperinflation and improved breathing control. For example, participants extend sung phrases with appropriate repertoire and repeat-voiced fricatives performed with biofeedback techniques, which aim to synchronise breath and phonation and match the work of the primary muscles of respiration in a way that supports appropriate vocal effort.^4^ ‘SLH’ repertoire intends to have therapeutic impacts, which includes a variety of voice qualities, extended vocal ranges, aiming to improve vocal efficiency and contribute to breath management strategies.^10^ These techniques are delivered musically and creatively within the context of promoting full body movement.

‘SLH’ programmes are commonly run at least weekly for at least 6 weeks. There is some evidence to suggest that SLH may be clinically effective—improving quality of life and functional capacity for individuals with COPD.^4 11–17^ However, large-scale randomised controlled trial (RCT) data are lacking and the overall research quality in the field has been low to very low as judged by a Cochrane systematic review.^11^ Current SLH RCTs in progress are investigating SLH compared with standard care in people with COPD (NCT04034212, ISRCTN42943709) or comparing singing as the physical training intervention in pulmonary rehabilitation with the current gold-standard aerobic and strength exercise training in people with COPD (NCT03280355). Multiple systematic reviews have highlighted the need to increase the evidence base for singing, both in relation to clinical effectiveness and underlying physiological impacts related to participation.^4 11^

This narrative review was produced by a range of allied health and medical professionals, a singing group leader and vocal coach with a special interest in the field of SLH. The review explores the physiological rationale for SLH as an intervention in COPD, aiming to highlight potential physiological mechanisms requiring further research, focusing on:

1. The physiology of breathing and singing.
2. Abnormalities of pulmonary mechanics in COPD.
3. Potential physiological impact of singing in people with COPD.

We use the term ‘may’ both to raise potential interesting hypotheses for future research in SLH and where uncertainties in the literature remain due to a lack of quality evidence.

## The physiology of breathing and singing

### Breathing control while singing

During quiet breathing, inspiration is an active process engaging the muscles of respiration, while expiration is passive, driven largely by lung elastic recoil. During physical activity, as metabolic demands rise, minute ventilation increases by increasing both tidal volume and respiratory rate. This requires increased flow rates and the recruitment of abdominal muscles to deliver active expiration. Regulation of the glottic aperture by laryngeal muscle activity also helps to control ventilation.^18^ The glottis widens on inspiration and narrows on expiration.^19^ Glottic opening occurs prior to the descent of the diaphragm on inspiration, and the glottis acts as a valve to influence the expiratory time of the respiratory cycle.^20^ Therefore, the larynx could be considered a key modulator of expiratory flow.^20^

During speech and singing, breath duration and flow rates are controlled to support sound generation by the larynx. Thus, inspiration and expiration are both active in order to adjust lung volumes for phrase length and sound volume.^21^ Herbst^10^ critiqued the traditional linear relationship of the power-source-filter model, where the sung voice is determined by the lungs and then modified by the larynx and vocal tract. Rather he states that each system and vocal subsystem has a physical effect on the other. In this regard, changes in vocal function will alter exhalation accordingly. Singers partly control exhalation through the activity of the abdominal muscles including the rectus abdominus, internal and external obliques and transverse abdominus.^21^ The internal intercostals also work to draw the ribcage during phonation.^21^ During speech, inspiratory time is reduced and expiratory time is lengthened (compared with a passive breathing cycle).^22^ Subglottic pressure is regulated more actively during speech and singing compared with unphonated breathing and even more so while singing.^23–25^

Phonation requires sustaining-controlled exhalation. In a study using electrical impedance tomography, Traser *et al*^26^ show that phonation modulates expiratory airflow in a more uniform pattern compared with unphonated breathing as phonation prevents the majority of air being exhaled at the start of the breath. Zhang^23^ reports that subglottal pressures can be maintained throughout exhalation with minimal glottal resistance. This results in the lung volume at the end of the phrase being close to the residual volume. This requires higher expiratory pressures following inspiratory pressures to change lung volumes at this point in lung capacity. The respiratory and vocal mechanisms work synergistically to optimise efficiency in the work of singing.

### Lung function parameters in singers

Total lung capacity depends on the balance between inspiratory muscle strength and the elastic recoil of the respiratory system as well as the individual’s size, sex, ethnicity and disease state. Respiratory muscle strength can be improved with specific training^27 28^ or as a result of pulmonary rehabilitation.^29^ Watson *et al*^30^ studied professional singers comparing singing and non-singing ventilatory tasks. The study found that vocalisation required activation of additional muscles, such as latissimus dorsi, compared with non-singing ventilatory tasks, which may be relevant in relation to respiratory muscle strength. Furthermore, singers have been shown to have forced expiratory volumes and vital capacities greater than population norms.^31–34^ Differences in lung function parameters between singers and non-singers likely result from multiple factors, including duration of singing participation^33^; a propensity for people with pre-existing above-average respiratory function to become singers as is known from other physically demanding professions; and, potentially, lifestyle choices including smoking less and exercising more compared with peers.^34^ However, it is speculated that repeated focus on controlled exhaled breath improves expiratory muscle strength.^32^

A small study of nine professional singers showed that they were able to dynamically alter vocal fold aperture and abdominal volumes to control their breath.^35^ Furthermore, a small study comparing seven classically trained singers to four untrained individuals suggested singers were able to substantially alter the coordination of abdominal and thoracic volume change during singing compared with quiet breathing, by using a greater percentage of abdominal contribution to lung volume change.^36^ Solomini *et al*^36^ comment that classically trained singers increase abdominal pressures to enable a more effective expiration by optimising muscular length-tension ratios and force-generation capacity. It is speculated that this is possible because during singing, the anterior diaphragm and ribcage, and the middle and posterior diaphragm and ribcage, act as different functional units. These separate units maintain subglottal pressures with changing recoil forces on expiration.^37^

During singing, abdominal muscle activity increases. Macdonald *et al*^38^ investigated the acute effects of singing on abdominal musculature in 25 healthy adults using ultrasound. During singing, the internal oblique and transverse abdominis (TA) muscles both contract and TA has a greater percentage contraction compared with baseline conditions at a comfortable inspiration. Salomoni *et al*^36^ used respiratory inductance plethysmography bands, showing that trained singers use a greater abdominal contribution to change lung volumes during singing compared with untrained controls. It is important to recognise that the majority of singing studies have been conducted on classically trained singers, whose breathing requirements are specific to the style that they sing, and different to contemporary pop or global repertoire, often used in SLH.

## Abnormalities in pulmonary mechanics in COPD

COPD encompasses pathologies including chronic bronchitis and emphysema, which alter pulmonary mechanics.^39^ Airflow obstruction occurs results from loss of small airways, airway inflammation and wall thickening, mucous hypersecretion, an impaired mucociliary escalator and airway muscle hypertrophy. Additionally, emphysema reduces lung elastic recoil, promoting premature airway closure and gas trapping. The combination of increased airflow resistance and increased lung compliance increases the time constant of lung units and leads to hyperinflation, which worsens with exercise.^40 41^ As operating lung volumes increase, the inspiratory muscles develop a mechanical disadvantage with a reduced force generation capacity because of the length–tension relationship as the diaphragm has a shorter, flatter position.^42 43^ Inspiratory reserve volume decreases as total lung capacity is approached and the flatter position on the pressure–volume relationship means that the work of inspiration increases. This leads to mechanical constraint on maximum ventilation, recruitment of accessory muscles of respiration, breathlessness and the premature termination of exercise. In addition, skeletal muscle weakness and loss of endurance are common and increase ventilatory demand during daily activities.^44–46^ Forty seven per cent of patients with COPD have been shown to have dysfunctional breathing, which is more severe and prevalent than in asthmatics or healthy controls.^47^ Furthermore, the Nijmegen Questionnaire score, a measure of hyperventilation syndrome (a type of dysfunctional breathing), is a strong independent determinant of disease-related quality of life in COPD as measured by the COPD Assessment Test.^48^ Both the Nijmegen Questionnaire and COPD Assessment Test measure shortness of breath and chest tightness, and to the authors’ knowledge, divergent validity between these measures has yet to be established, which is a potential limitation to Brien *et al*’s study.^48^ Nevertheless, the findings from both Law *et al*^47^ and Brien *et al*^48^ indicate that breathing pattern retraining is indicated in COPD populations. Breathing pattern retraining has shown to improve physiological outcomes for individuals with COPD, including improvements in functional exercise capacity, respiratory pattern, respiratory rate, lung function, oxygenation, a reduction in dynamic hyperinflation on exertion and functional anatomical dead space volume.^49–55^

## Potential physiological impact of singing in people with COPD

There is a clear physiological link between the upper airway and lower airway, ventilation and voice quality.^10^ It has been stated that dysphonia in COPD can be functional in origin and, therefore, corrected somewhat with therapy.^56^

Techniques that slow down expiration allow a greater degree of lung emptying, which in turn lowers operating lung volumes and can make breathing more comfortable for individuals living with COPD.^57^ Many breathing retraining techniques focus on slowing down exhalation. Singing activities can be adapted and performed in a similar fashion. In addition, singing may strengthen musculature, improve posture and work as an exercise modality as a structured, repetitive and goal-directed activity, all with potential impacts on symptoms. We explore such possibilities below.

Singing was first documented as a potential therapeutic intervention for individuals with COPD 35 years ago,^58^ where it was suggested that singing enabled greater expectoration of sputum and improved blood gasses when the patients took larger breaths to sing. Goldenberg^59^ has previously discussed how the increase in airway shear forces and oscillatory action of singing may help expectorate sputum. A further potential therapeutic mechanism is the alteration of breathing patterns for people living with COPD, which has potential clinical utility.^50^ Binazzi *et al*^60^ investigated the breathing pattern of people with COPD singing a Christmas carol using optoelectric plethysmography. None of the participants in the study had professional or amateur singing experience. Individuals listened to the Italian version of ‘O Christmas Tree’ once before performing all four verses three times—the third attempt used for analysis. The authors report singers adopted higher operating volumes to maintain adequate expiratory flow generation. Female singers showed greater volume changes in their chest wall, whereas male singers sung with greater abdomen volume changes compared with quiet breathing. In this study, singing altered breathing patterns in patients with COPD and there was wide variability in measured end expiratory chest wall volumes between individuals from a small sample size. It is not known whether the different volume changes between sexes was driven by different body shapes or different keys and registers used in singing the chosen repertoire.

Given that singing requires participants to consciously modulate their breathing patterns, it is plausible that appropriately selected singing repertoire and vocalisation tasks could be used to enable breathing pattern optimisation. Lord *et al* studied weekly ‘SLH’ training for 126 and 8 weeks.^13^ Primary outcome measures relating to breath control^12^ and physical activity^13^ were not met. Though of note, the breath control measures were developed to assess hyperventilation rather than breathing control in COPD; therefore, their value in this context is questionable. However, concerning secondary outcome measures, both studies identified improvements in the physical component score of the SF-36 health status assessment tool. As discussed previously, improving respiratory strength could be useful for people with COPD, and inspiratory muscle strength has been shown to improve with singing for a group of older people, of whom a quarter were living with ‘respiratory disease’.^61^ However, further research including individuals diagnosed with COPD is warranted.

An RCT in Brazil, comparing 24 group singing sessions to 24 handcraft work sessions for people with COPD,^62^ suggested singing group participants experienced reduced dyspnoea and improved oxygen saturations during singing. However, these changes did not appear to be sustained beyond the end of the 24-week programme.

The perception of severity of breathlessness does not always match the degree of airway pathophysiology in COPD.^63^ Herigstad *et al*^64^ demonstrated significant correlations between improvements in how breathless and anxious patients felt following a course of pulmonary rehabilitation and the brain activity of multiple regions according to functional MRI, suggesting a potential role for modifications to interoception. They suggest that becoming breathless in a safe space under supervision may alter the perceptions of breathlessness. Approaches, such as SLH, focus on creating safe, supervised spaces, which may help to explain changes in breathlessness reported by SLH participants. The moderate physical activity of SLH is not only enjoyable, but one where the breath is used to create sound in music collaboratively within a group. The result of such activity can be profound: ‘When we sing, the breath enables creation of something new, promoting life not inhibiting it. It is the barrier that is forgotten, not the breath’.^8^

Posture and the voice are coordinated during vocal effort, particularly with the correlation of forward sagittal movement of the trunk at increased sound pressure levels.^65^ The ability for the diaphragm to act as a postural control muscle within the trunk is reduced in situations where individuals have increased neural respiratory drive, dead space ventilation and simultaneous arm movements.^66^ Evidence suggests that singing challenges postural control and singing training improves postural control.^67^ In the study by Peultier-Celli *et al*,^67^ professionally trained singers challenged their balance by standing on a force plate in various conditions, including eyes-open, eyes-closed and singing chosen arias. Singers’ sway was greater when singing compared with standing with eyes open. However, better postural control was observed in those who had more years of singing experience. ‘SLH’ actively trains postural control to support singing. One of the 10 main competencies suggested for trained singing leaders is that they have ‘a holistic understanding of the kinaesthetics and whole body proprioception in breathing’.^9^ It is not yet certain how SLH affects posture, but qualitative reports indicate that posture improves through participation^12^ with pilot quantitative data, suggesting improvements to balance confidence.^68^

‘SLH’ exercises have been compared with different speeds of treadmill walking in a small study in healthy individuals showing that singing is associated with a similar metabolic demand to moderately vigorous physical activity.^69^ This study also demonstrated that tidal volumes during singing were greater than those performed for treadmill walking matched for physical activity intensity (Mean Difference: 1.18 L p=0.01), which is likely to relate to the additional volume of air needed for phonation in addition to metabolic requirements, and the phrasing of singing components which necessitated reduced breathing frequency compared with what might have been taken naturally. Further research is required to see if this is reproduced in older people and in people with COPD.

Liuzijue Qigong is a form of traditional Chinese martial art characterised by breathing, which focuses on the use of the primary muscles of respiration (diaphragmatic breathing), pursed-lip breathing and the production of different sounds during limb movements.^70^ The physical and vocal warm-up exercises of ‘SLH’ are similar exercises to Liuzijue Qigong. Liuzijue Qigong has been shown to improve maximal inspiratory and expiratory pressures and specific airway conductance in patients with COPD.^70–72^ The sounds produced in Liuzijue Qigong are similar to the voiced fricatives used as vocal warm-ups in SLH. Similar to Liuzijue Qigong, many repertoire choices in SLH include long phrases with open vowel sounds. Long phrases and open vowel sounds have been beneficial in reducing respiratory rate and increased heart rate variability via toning and repeated mantra singing.^73–75^ Further research is needed to determine the physiological sustained effects once the singing stops. ‘SLH’ exercises also focus on semiocclusion for extended breaths, which also aim to control and support exhalation. ‘SLH’ leaders receive vocal training and toolkits prior to running groups. It is the skill of the trained singing leader to use the wide-ranging research-based techniques sensitively, within the flow of musical activity to engage individuals, making the experience fun and offer a perceived non-clinical environment, which promotes self-expression. The weekly repetition of exercises and games aim to reinforce the ability to use these techniques independently as self-management strategies.

Table 1 details the actions of singing and the potential mechanisms of benefit for individuals with COPD:

**Table 1:** Singing actions and proposed/potential physiological basis of benefits for individuals with COPD

Singing action	Proposed benefits for individuals with COPD
Expiration during singing requires controlled muscle activity	Controlling expiratory flow,^20 37^ lower operating lung volumes and make breathing more comfortable.
Regulation of glottic aperture and improved glottic efficiency	Controlling of expiratory flow.^20^
Alters abdominal and thoracic contribution to lung volume changes.	Doming the diaphragm supports exhalation, also improving length-tension ratio and force-generation capacity on inspiration.^36 37^
Inspiratory muscle training	Greater inspiratory strength is required to generate higher pressures to inspire at large lung volumes (hyperinflation).^78^ Facilitates active phases of breathing, when required.
Enhancing airway shear forces	Potential to promote airway clearance, reduces symptom burden^59^
Training of posture and balance	Optimises lung function, potential to reduce risk of falls and increase balance confidence^68^
Singing is a moderately intense physical activity (shown specifically with core components of SLH)	Increasing physical activity levels^69^ in a population significantly physically inactive^79^

COPD, chronic obstructive pulmonary disease; SLH, Singing for Lung Health.

Table 1 highlights potential physiological benefits of singing for individuals with COPD. However, due to both the lack of high-quality evidence and relatively few studies specific to singing training which is not classically derived or studies specific to SLH, more definitive associations and causality statements cannot currently be made between physiological studies in COPD, singing and the practice of SLH. The findings from small studies in classically trained singers to date have limited external validity compared with the breath management required from different repertoire and training, such as pop singing, where singers move from speech, to belt-type singing and falsetto. The breath demands will vary accordingly. Herbst^10^ provides a good review of how different voice subsystems contribute to breath support, which we do not cover in this review.

Recommendations from previous systematic reviews remain,^4 11^ where more research is required in order to establish the clinical effectiveness and physiological mechanisms underpinning any beneficial effects achieved through SLH participation. Table 2 provides some research questions and types of clinical studies which are now required in order to further explore the physiological mechanisms of potential benefit and clinical effectiveness of SLH:

**Table 2:** Physiological research questions and proposed clinical effectiveness studies required in SLH

Physiological research questions	Clinical effectiveness studies
Do SLH techniques and participation in SLH groups change sputum expectoration volume, cough and exacerbation frequency?	SLH group participation vs generic community choir participation.
How do different SLH techniques change laryngeal function and airflow compared with passive breathing?	SLH group participation as a maintenance intervention post-PR completion vs standard care.
How do SLH techniques change respiratory and abdominal muscle activity compared with passive breathing?	SLH as one-to-one inpatient and outpatient option vs traditional physiotherapy for symptom management (breathlessness, airway clearance).
How do SLH techniques change thoracoabdominal volumes compared with passive breathing?	Online SLH group participation vs face-to-face group participation.
How does participation in SLH groups change posture and balance?	Post-exacerbation SLH vs standard care.
Does long term participation in SLH groups alter the trajectory of lung function decline in COPD?	PR vs SLH for those who decline PR.
What level of physical activity is performed by individuals in a SLH group?	SLH vs usual care in different settings and populations.
Does participation in SLH groups improve inspiratory and expiratory muscle strength?	
Is there a difference in breathing pattern between SLH repertoire directed performance and other repertoire such as pop and classical?	

COPD, chronic obstructive pulmonary disease; PR, pulmonary rehabilitation; SLH, Singing for Lung Health.

This review is specifically focused on physiological rationale and outcomes. However, we acknowledge that SLH is an intervention, which aims to provide holistic health benefit. Any health-related benefits, resulting from participation, are likely multifactorial with psychological and social components, which are known to be important in COPD.^12 16 68 76 77^ Further research should continue to use a range of biopsychosocial outcomes in order to evaluate the different mechanisms of benefit effectively.

## Conclusion

Breathing and singing are intimately related. There is a good theoretical rationale to support the therapeutic use of singing for people with COPD as a method of improving physiological parameters and breath control; however, the research in this area is limited and of generally low quality. Further research is required to more effectively assess the impact of SLH for people with COPD, and which physiological mechanisms underlie any improvements observed.

## Data Availability

No data are available. Not applicable.
